# Cuproptosis-related molecular alterations in a neonatal rat model of hypoxic–ischemic brain damage

**DOI:** 10.3389/fneur.2026.1855158

**Published:** 2026-09-23

**Authors:** Qiqi Gao, Yu Fang, Tong Lou, Junyi Qian, Yu Chen, Wei Hu, Zhaochun Peng, Hang Zhang, Zhenlang Lin, Wei Lin

**Affiliations:** Department of Pediatrics, The Second Affiliated Hospital and Yuying Children's Hospital of Wenzhou Medical University, Wenzhou, Zhejiang, China

**Keywords:** cuproptosis, hypoxic–ischemic brain damage, mitochondrial damage, neonatal rat, oxidative stress

## Abstract

**Background:**

Copper homeostasis imbalance is closely linked to the progression of various neurological disorders. Cuproptosis, a recently identified form of cell death caused by abnormal intracellular copper accumulation, has an unclear relationship with hypoxic–ischemic brain damage (HIBD). This study aims to characterize cuproptosis-associated molecular changes in the pathological process of HIBD in neonatal rats.

**Methods:**

A neonatal rat HIBD model was established using the modified Rice–Vannucci method. Cerebral blood flow was measured with laser Doppler flowmetry, while infarct volume was assessed through 2,3,5-triphenyltetrazolium chloride staining. To evaluate brain tissue damage and mitochondrial ultrastructural changes, histological staining and transmission electron microscopy were utilized. Specific assay kits were used to detect oxidative stress markers and copper ion levels in the brain tissue. Immunofluorescence staining was employed to monitor DLAT expression in neural cells, and Western blotting was conducted to analyze the expression of cuproptosis-related proteins.

**Results:**

Compared to the Sham group, the HIBD group exhibited significantly reduced perfusion in the injured cerebral hemisphere, which was associated with liquefactive necrosis and tissue atrophy. Additionally, increased levels of copper ions and ROS, along with decreased levels of GSH and GPX4, indicated that HIBD leads to copper dyshomeostasis and oxidative stress. Staining results showed glial cell activation, neuronal loss, and widespread downregulation of DLAT expression in the HIBD group, suggesting the presence of neuroinflammatory responses and cellular metabolic disturbances. Furthermore, the upregulation of cuproptosis-related proteins FDX1 and HSP70, along with the downregulation of LIAS, ACO2, and Lip-DLAT, and such molecular changes correlate with impaired lipoylation metabolism and iron–sulfur cluster biosynthesis, aligning with molecular signatures of cuproptosis.

**Conclusion:**

This study identifies multiple cuproptosis-related molecular alterations coinciding with neuronal injury after HIBD in neonatal rats; these changes are accompanied by impaired lipoylation modification and disrupted redox homeostasis.

## 1 Introduction

Neonatal hypoxic–ischemic brain damage (HIBD) is a serious neurological condition caused by reduced cerebral blood flow and oxygen deprivation. It is a leading cause of neonatal mortality and can lead to long-term neurological impairments (1). Among survivors, 5–10% of infants exhibit persistent motor deficits, while 20–50% continue to experience sensory or cognitive abnormalities into adolescence (2). However, the pathogenesis of neonatal HIBD remains complex, and clinical treatment options are relatively limited (3), underscoring the importance of further investigating its underlying mechanisms.

Copper is an essential metal ion necessary for normal brain development, and maintaining its homeostasis is crucial for supporting neuronal physiological activities. The entry of copper into the central nervous system depends on brain barrier systems, which include the blood–brain barrier (BBB) that separates blood from brain interstitial fluid and the blood–cerebrospinal fluid barrier (BCB) that separates blood from cerebrospinal fluid (CSF) (4). Copper is mainly stored in glial cells and is released dynamically through calcium-dependent secretory pathways in response to the needs of neurons (5). Within neurons, copper acts as a key cofactor in multiple enzymatic processes, including cytochrome c oxidase (COX)-mediated mitochondrial energy metabolism (6), superoxide dismutase (SOD)-related antioxidant defense (7), and dopamine β-hydroxylase (DBH)-catalyzed neurotransmitter synthesis (8). The brain, with its high metabolic activity and numerous copper-dependent enzymes, is especially susceptible to disruptions in copper homeostasis (9).

Cuproptosis is a newly recognized type of regulated cell death caused by excessive accumulation of intracellular copper, setting it apart from other known cell death pathways. When there is an overload of copper, copper ions directly interferes with the tricarboxylic acid (TCA) cycle in the mitochondrial respiratory chain, which triggers the oligomerization of lipoylated proteins and destabilization of iron–sulfur cluster (ISC) proteins; these alterations subsequently induce proteotoxic stress and oxidative damage, ultimately culminating in cell death (10). Growing evidence indicates a strong link between copper dyshomeostasis and the pathogenesis of HIBD. In HIBD patients, serum copper levels are significantly higher than those in healthy controls (11). Hypoxia upregulates the expression of the high-affinity copper transporter 1 (CTR1), enhancing cellular copper uptake (12), while ischemia-induced reactive oxygen species (ROS) can disrupt copper-binding protein structures, promoting their degradation and the release of free copper ions (13). In cerebral ischemia models, the copper chelator tetrathiomolybdate (TTM) significantly reduces infarct volume and enhances antioxidant capacity, effects that are attributed to its modulation of oxidative stress and inflammatory responses (14). While these studies emphasize the critical role of copper imbalance in HIBD, direct evidence of cuproptosis in its pathological process is still lacking. To address this gap, we established a neonatal rat HIBD model to examine the expression of cuproptosis-related molecules and characterize cell death phenotypes. The goal is to preliminarily characterize molecular alterations linked to cuproptosis in the pathogenesis of HIBD.

## 2 Materials and methods

### 2.1 Experimental animal husbandry

All animal care and experiments were conducted strictly following the Guide for the Care and Use of Laboratory Animals and were approved by the institutional ethics committee of the Wenzhou Institute, University of Chinese Academy of Sciences (Approval No. WIUCAS24010903). Adult Sprague–Dawley (SD) rats (weight 200–300 g) were purchased from Zhejiang Weitong Lihua Test Animal Technology Co., Ltd. and were kept in an specific pathogen-free (SPF) animal breeding room of the Animal Laboratory Center, which was maintained at a temperature of 23 ± 2 °C, a humidity of 60 ± 10%, and a 12-h light–dark cycle. Male and female rats were allowed to mate freely at a ratio of 2:1, and the pregnant females were kept in separate cages. The neonatal rats had unrestricted access to their mothers, recorded as P0 on the day of birth. All solid granulated feed and water were sterilized. Neonatal pups from multiple litters were randomly allocated to Sham or HIBD groups to minimize litter-bias effects. Animals that exhibited surgical mortality, obvious poor physical condition, or failed model establishment were pre-defined as exclusion criteria. Mortality and model-failure rates were recorded during the experiment. Where feasible, investigators were blinded during outcome assessment. At all experimental endpoints, neonatal rats were euthanized by overdose of inhaled isoflurane (5%). Complete loss of pedal withdrawal reflex was confirmed before tissue collection.

### 2.2 HIBD model

In this experiment, the HIBD model of neonatal rats was established by using the modified Rice-Vannucci method (15). Male SD rats at 7 days old (P7) were anesthetized with isoflurane (3–4% for induction, 1–2% for maintenance) and secured on the animal operating table. After disinfection, a 3–5 mm surgical incision was made along the midline of the neck. Under a dissecting microscope, the proximal and distal ends of the left common carotid artery were ligated and separated to obstruct blood flow. The rats were then placed on a heating pad for 30 min to recover before being transferred to a hypoxic chamber (92% nitrogen, 8% oxygen) at 37 °C for 2 h. After this period, they were returned to their mothers. In contrast, rats in the Sham group were only exposed to the left common carotid artery without undergoing ligation or hypoxic treatment.

### 2.3 Laser speckle contrast imaging (LSCI)

LSCI (RFLSI III, RWD Life Science Co., Ltd., Shenzhen, China) is a hemodynamic imaging technique used to observe and measure blood flow dynamics in living tissues. To evaluate the degree of ischemic injury, we compared the regional cerebral blood flow (rCBF) differences between the bilateral hemispheres of two groups of rats. After 24 h of modeling, P8 rats were anesthetized using isoflurane inhalation and then positioned prone on a 37 °C thermostatic surgical pad to maintain a constant body temperature. A longitudinal incision was made along the midline of the scalp to fully expose the parietal bone. The head was then positioned within the sampling area, and a 785 nm wavelength laser beam was employed to measure blood flow in the lateral cerebral hemisphere. Normal saline was intermittently applied to keep the skull surface moist during the measurement. Laser speckle images and relative cerebral blood flow (CBF) values in the region of interest (ROI) were analyzed using LSCI software (RWD Co., Ltd.).

### 2.4 2,3,5-Triphenyl tetrazolium chloride (TTC) staining

TTC staining was employed to identify ischemic infarcts in mammalian tissues. After 24 h of modeling, P8 rats were anesthetized with isoflurane and rapidly decapitated. The isolated brain tissue was frozen at −80 °C for approximately 5 min and subsequently sectioned into 5–6 coronal slices at 2-mm intervals. The brain slices were then tiled and immersed in a 2% TTC solution (G3005, Solarbio Science & Technology Co., Ltd., Beijing, China). They were stained in a 37 °C incubator, kept in the dark for 30 min, and shaken periodically. The degree of ischemia in the brain slices was assessed: viable tissue stained red, whereas infarcted (ischemic necrotic) tissue appeared pale or white. ImageJ software (Ver. 1.53e; National Institutes of Health, Bethesda, MD, USA) was utilized for analysis. The percentage of infarct volume in the brain tissue was calculated using the formula: (sum of ischemic volumes of each slice)/(sum of volumes of each slice) × 100%.

### 2.5 Brain tissue water content detection

The water content of brain tissue was measured to assess the degree of brain edema. After 24 h of modelling, P8 rats were anesthetized with isoflurane, and the brain tissue from the left side (the injured side) was quickly excised and weighed (wet weight). The brain tissues were then placed in an oven at 100 °C for 72 h, after which they were removed and weighed again (dry weight). Brain water content was calculated using the following formula: brain water content = (wet weight − dry weight)/wet weight × 100%.

### 2.6 Histological staining

Seven days after modelling, P14 rats were anesthetized with isoflurane and underwent transcardial perfusion with 10 mL of normal saline followed by 10 mL of 4% paraformaldehyde (PFA). Brain tissue was then harvested and post-fixed in 4% PFA at 4 °C overnight. The anterior and posterior one-thirds of the brain tissues were trimmed, and the remaining tissue was embedded in paraffin blocks after undergoing gradient ethanol dehydration and xylene clearing. These paraffin blocks were cut into 5 μm thick coronal sections. The sections were deparaffinized, hydrated, and stained using hematoxylin–eosin (HE) (G1120) and Nissl (tar violet method) (G1430) staining kits, all obtained from Solabio. Images were observed and captured using a light microscope (Eclipse NI-U, Nikon Co., Ltd., Tokyo, Japan).

### 2.7 Immunofluorescence staining

Paraffin-embedded brain sections were dewaxed, rehydrated, and repaired using a citrate repair solution (C1010, Solarbio) through high-temperature heating. After cooling to room temperature, the sections were blocked with 10% normal goat serum for 30 min and then incubated overnight at 4 °C with primary antibodies against glial fibrillary acidic protein (GFAP) (1:1000, AFRM9172), ionized calcium-binding adapter molecule 1 (IBA-1) (1:1,000, AFRM0011), neuronal nuclei (NeuN) (1:1000, AFRM9616) (from Aifang Biotechnology Co., Ltd., Hunan, China), and dihydrolipoamide S-acetyltransferase (DLAT) (1:200, A14530, ABclonal Technology Co., Ltd., Wuhan, China). The samples were subsequently warmed to 37 °C for 30 min, washed, and incubated with appropriate fluorescent secondary antibodies for 2 h at room temperature. After washing the brain sections, they were sealed with a 4′,6-diamidino-2-phenylindole (DAPI)-containing anti-fluorescence attenuation sealing tablet (S2110, Solarbio) and finally observed and photographed using a fluorescence microscope (Nikon Eclipse Ni-U).

### 2.8 Transmission electron microscopy (TEM) detection

At 24 h post-HIBD modeling, P8 rats were euthanized, and brains were rapidly harvested. The left cortical region was dissected and cut into 1 mm^3^ tissue blocks, which were fixed in pre-cooled glutaraldehyde at 4 °C for 2 h. After fixation with osmic acid, the tissue blocks underwent dehydration through an alcohol gradient and were then embedded osmotically. These blocks were subsequently cut into ultrathin sections measuring 60 nm in thickness. The sections were double-stained with 2% uranyl acetate and lead citrate before being air-dried at room temperature. The morphology of nerve cells was examined and photographed for preservation using TEM (Tecnai G20 TWIN, FEI Company, Hillsboro, OR, USA).

### 2.9 Specific detection kit

At 24 h post-HIBD modeling, P8 rats from both groups were euthanized, and the left brain tissues were dissected. Tissues were homogenized, centrifuged, and the supernatant was collected for analysis. ROS (A107976), glutathione (GSH) (A108057), and glutathione Peroxidase 4 (GPX4) (A129631) levels were quantified using commercial test kits (all from Affandi, Shanghai, China) following the manufacturer’s protocols. Standard solutions were serially diluted as instructed, while samples were diluted five-fold using the specimen diluent. Next, 50 μL of each standard or sample was dispensed into the microplate wells, which were then sealed and incubated at 37 °C for 30 min with gentle shaking. After discarding the liquid, the wells were washed five times with 300 μL of wash buffer, allowing a 30-s dwell time for each wash. Following this, 50 μL of enzyme conjugate was added to all wells except the blanks, and the plates were incubated again at 37 °C for 30 min. The washing procedure was repeated, after which color developer A and B were added sequentially. The plates were then incubated for 5 min at 37 °C, and the reaction was terminated by adding 50 μL of stop solution. For all three ELISA assays, sample concentrations in homogenate supernatant were calculated against standard curves fitted by four-parameter logistic regression. Final data are expressed as IU/mL (ROS), ng/L (GSH), and pmol/mL (GPX4). The optical density (OD) was read at 450 nm on a microplate reader (Thermo Scientific Varioskan LUX, Thermo Fisher Scientific Inc., Waltham, MA, USA).

### 2.10 Copper assay

The Copper (Cu) Colorimetric Assay Kit (E-BC-K300-M, Elabscience, Wuhan, China) was used for copper quantification. This colorimetric complex-formation assay measures total copper in bulk brain tissue homogenates, with a linear detection range of 1.84–60 μmol/L. Tissue copper concentrations were normalized to total protein content determined by the BCA assay. 24 h after modeling, P8 rats from both groups were euthanized, and left-sided brain tissues were harvested. The tissues were homogenized and centrifuged, and the supernatants were collected for analysis. In each well, 15 μL of either standard or sample was added, followed by 230 μL of chromogenic reagent. The plate was then sealed and incubated at 37 °C for 5 min. Finally, the OD values were measured at 580 nm using a microplate reader.

### 2.11 Western blotting (WB)

Following the establishment of the HIBD model at 24 h, P8 rats from both groups were sacrificed under deep isoflurane anesthesia. Brain tissues were rapidly collected post-anesthesia. Radio-immunoprecipitation assay buffer (RIPA) lysis buffer (P0013B, Beyotime Institute of Biotechnology, Shanghai, China) was added to the samples. After homogenizing the tissue and centrifuging the mixture, the supernatants were collected, and protein concentrations were measured using an enhanced bicinchoninic acid (BCA) protein assay kit (P0010, Beyotime). Subsequently, protein samples of varying molecular weights were separated by 8–12% sodium dodecyl sulfate-polyacrylamide gel electrophoresis (SDS-PAGE) and transferred to polyvinylidene fluoride (PVDF) membranes (IPVH00010, Millipore, USA). The membranes were then blocked with 5% skim milk in a shaking incubator at room temperature.

After washing with tris-buffered saline and tween 20 (TBST), membranes were incubated overnight at 4 °C with primary antibodies on a rocking platform. The primary antibodies used were: beta-actin (β-actin, 43 kDa, 1:20,000, 66,009-1-lg, Proteintech), aconitase 2 (ACO2, 85 kDa, 1:5,000, A3716, ABclonal), DLAT (70 kDa, 1:2,000, A14530, ABclonal), heat shock protein 70 (HSP70, 70 kDa, 1:1,000, A20819, ABclonal), ferredoxin 1 (FDX1, 14 kDa, 1:5,000, ab108257, Abcam), lipoyl synthase (LIAS, 42 kDa, 1:5,000, ab246917, Abcam), and lipoic acid (recognizes protein-covalently bound lipoyl moiety, 1:5,000, ab58724, Abcam). After washing the strips three times with TBST, membranes were incubated with corresponding secondary antibodies (goat anti-mouse, 1:10,000, SA00001-1, Proteintech; goat anti-rabbit, 1:10,000, AS014, ABclonal) for 2 h at room temperature. Antibodies were selected according to the datasheets provided by respective vendors; their specificity has been reported in previously published studies using the same catalog numbers. Protein bands were visualized using the ChemiDoc-XRS + imaging system (Bio-Rad Laboratories, Inc., Hercules, CA, USA); exposure times ranged from 10 s to 120 s depending on signal intensity. Densitometric analysis was performed using Image Lab software (Ver. 6.1; Bio-Rad Laboratories, Inc., Hercules, CA, USA) and ImageJ software.

### 2.12 Statistical analysis

Quantitative data are presented as mean ± standard error of the mean (SEM), with individual data points plotted in figures. Sample sizes were chosen based on previous similar neonatal HIBD studies. For within-animal comparison between left and right cerebral hemispheres from the same rat, a paired Student’s t-test was applied. Comparisons between independent Sham and HIBD groups were performed using unpaired Student’s t-test or Mann–Whitney U test as appropriate. Given the limited sample size in some experiments, normality testing was interpreted with caution. Each result is based on at least three independent experiments. All statistical analyses were conducted using GraphPad Prism (Ver.10.4.1; GraphPad Software Inc., San Diego, USA). A *p*-value < 0.05 was considered statistically significant.

## 3 Results

### 3.1 HIBD model establishment

To assess the successful establishment of the HIBD model, we dynamically monitored cortical CBF using LSCI after modeling. We compared rCBF between the bilateral hemispheres. The results revealed that the blood flow signal intensity in the left hemisphere of the HIBD group was significantly lower than that in the left hemisphere of the Sham group. Importantly, there was no significant difference in blood flow signal intensity between the two hemispheres in the Sham group. In contrast, the HIBD group showed a marked reduction in blood flow signal intensity in the left hemisphere compared to the right (Figures 1A–D). These findings indicate that common carotid artery ligation followed by systemic hypoxia significantly impaired cerebral blood perfusion in neonatal rats.

**Figure 1:**
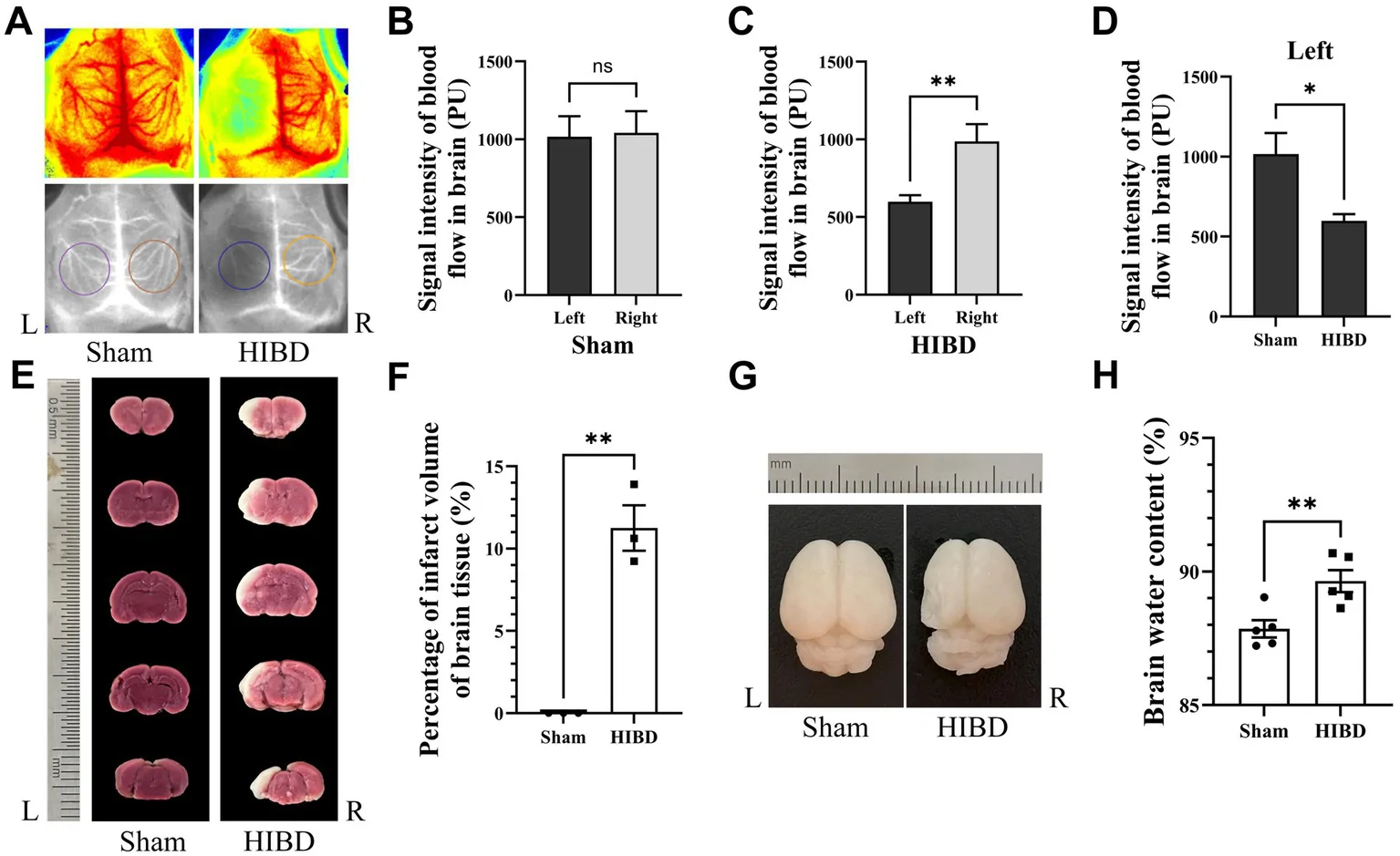
Hypoxia-ischemia causes significant brain damage. **(A)** Representative CBF images of the brain cortex and corresponding physical brain maps of rats 24 h after HIBD modeling. The ROIs used for the CBF assessment are outlined in circles in the images. **(B)** Two groups of blood flow signal intensity histograms. **(C)** Histogram of blood flow signal intensity on the left and right sides of the Sham group. **(D)** Histogram of blood flow signal intensity on the left and right sides of the HIBD group. **(E)** Representative results of TTC staining in coronal brain sections 24 h after HIBD modeling. **(F)** Percentage histograms of infarct volume of brain tissue in each group. **(G)** Representative images of the anatomical structure of the rat brains P14 after modeling. **(H)** Histogram of the brain water content of each group 24 h after HIBD modeling. The values represent the mean ± SEM, *n* = 10 for **(B–D)**; *n* = 3 for **(F)**; *n* = 5 for **(H)**. *p < 0.05, **p < 0.01 designate significant differences. The ns designates no significant difference. Scale bar = 1 cm. L, Left; R, Right.

Brain tissue infarction was evaluated using TTC staining, which showed uniformly red staining in the Sham group brains. In contrast, the left cortex of the HIBD group brains exhibited distinct pallor (Figure 1E). Quantitative analysis of infarct volume further confirmed significant cerebral infarction in the injured hemisphere (Figure 1F). Additionally, comparisons of brain water content and morphological examinations revealed liquefaction, necrosis, and atrophy in the injured hemisphere of the HIBD group, accompanied by a significant increase in brain water content compared to the Sham group (Figures 1G,H). These findings indicate that hypoxic–ischemic insult severely compromised brain tissue integrity, thereby confirming the successful establishment of the neonatal rat HIBD model.

### 3.2 HIBD induces pathological changes and neuronal injury in brain tissues

The hippocampus, a crucial brain region involved in learning, memory, and other functions, is particularly susceptible to brain injury. This vulnerability is due to the high sensitivity of its cornu ammonis 1 (CA1), cornu ammonis 3 (CA3) subregions, and dentate gyrus (DG) to hypoxia and excitotoxicity. HE staining revealed that the cerebral cortex and hippocampal subregions in the Sham group maintained intact tissue structure and orderly cellular arrangement. In contrast, the HIBD group exhibited significant widening and loosening of the cerebral cortical tissue, disordered cell arrangement in the hippocampal CA1 and CA3 subregions, and noticeable cell loss in the DG. These morphological observations show prominent histopathological changes following HIBD (Figure 2A).

**Figure 2:**
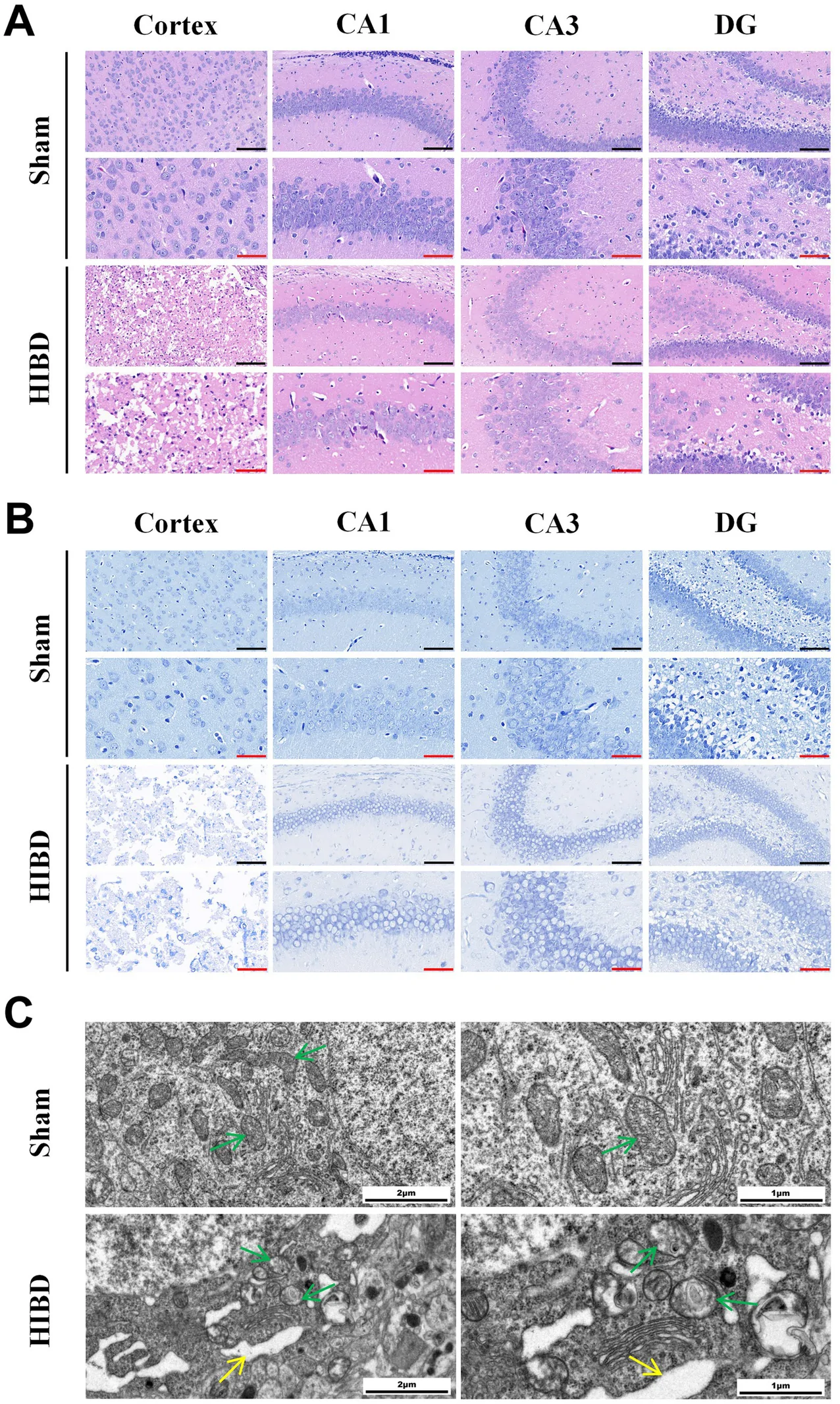
Structural damage of brain tissue of rats after HIBD. **(A)** Representative HE staining images of left cortical and hippocampal CA1, CA3 and DG regions P14 after HIBD modeling. Black scale bar = 100 μm, red scale bar = 50 μm. **(B)** Representative Nissl staining images of left cortical and hippocampal CA1, CA3 and DG regions P14 after HIBD modeling. Black scale bar = 100 μm, red scale bar = 50 μm. **(C)** Representative TEM images of different groups of cerebral cortex on the modeling side 24 h after modeling. The green arrows indicate mitochondria and the yellow arrows indicate endoplasmic reticulum. Scale bar = 2 μm, or 1 μm as indicated.

Nissl staining further illustrated that the Sham group contained abundant and deeply stained Nissl bodies across all brain regions, reflecting a healthy functional state of neurons. Conversely, reduced Nissl-body signals were observed in the cerebral cortex and hippocampal subregions of the HIBD group, pointing toward compromised neuronal synthetic function (Figure 2B). TEM observations revealed that neurons in the Sham group possessed mitochondria with regular morphology and clear cristae. In the HIBD group, however, mitochondria displayed diffuse swelling, cristae fragmentation and lysis, while the endoplasmic reticulum was severely dilated into vacuoles, indicating significant damage to subcellular structures (Figure 2C). Collectively, these results suggest that hypoxia-ischemia leads to notable neuronal injury across multiple brain regions.

### 3.3 HIBD induces increased oxidative stress and copper ion accumulation in brain tissues

Cuproptosis is closely linked to an imbalance in intracellular oxidative stress and disorders in copper ion metabolism. Kit-based detection indicated that, compared to the Sham group, the level of ROS in the ischemic brain tissues of rats in the HIBD group was significantly increased, while the levels of GSH and GPX4 were significantly decreased. These results imply that HIBD leads to elevated oxidative stress levels and impaired antioxidant defense mechanisms in brain tissues (Figures 3A–C). Meanwhile, a colorimetric assay for copper concentration revealed that the copper ion content in the ischemic brain tissues of the HIBD group was significantly higher than that in the Sham group, suggesting that hypoxic–ischemic injury can cause abnormal accumulation of copper ions in brain tissues (Figure 3D). Together, these findings indicate a clear imbalance in oxidative stress and copper ion metabolism during HIBD in neonatal rats. This provides important evidence for the potential role of cuproptosis in brain tissue injury following HIBD.

**Figure 3:**
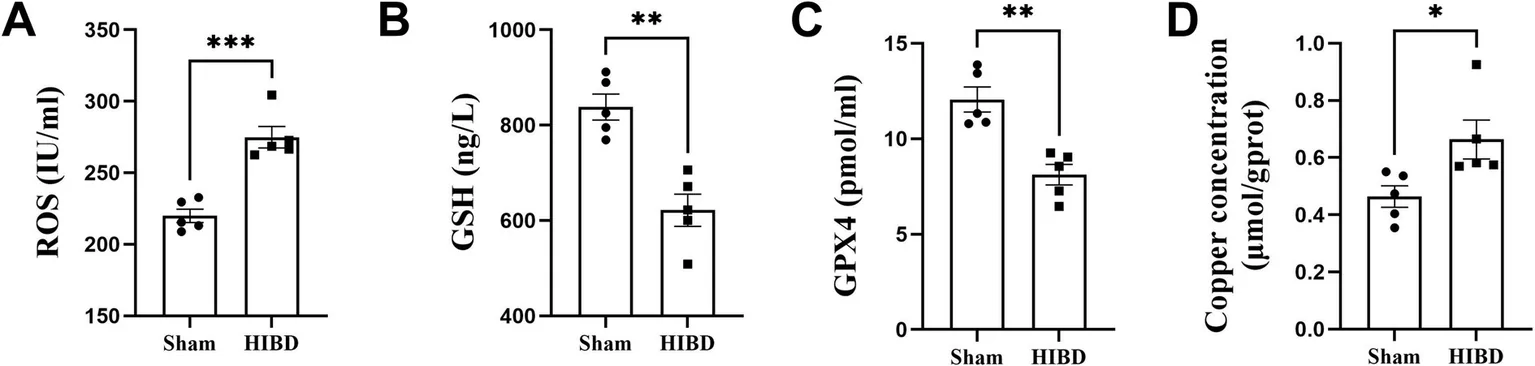
Changes in oxidative stress indicators and copper ion content in brain tissues of rats after HIBD. **(A)** Detection results of the ROS-specific detection kit. **(B)** Test results of the GSH-specific detection kit. **(C)** Test results of the GPX4 specific detection kit. **(D)** Test results of the copper ion-specific detection kit. The values represent the mean ± SEM, *n* = 5. *p < 0.05, **p < 0.01, ***p < 0.001 designate significant differences.

### 3.4 Expression characteristics of DLAT in different neural cell types after HIBD

Cuproptosis is strongly linked to abnormalities in key mitochondrial metabolic enzymes. DLAT, an essential enzyme in the mitochondrial TCA cycle, is believed to play a role in post-HIBD neuronal injury through functional disorders. In this study, we performed immunofluorescence staining to examine the co-expression of the microglial marker IBA-1, the astrocyte marker GFAP, the neuronal marker NeuN, and DLAT in the left cerebral cortex of rats from both groups. The results indicated that the HIBD group exhibited significant histopathological changes and differences in expression compared to the Sham group (Figure 4). The increased fluorescence intensities of IBA-1 and GFAP suggested the activation and proliferation of microglia and astrocytes. In contrast, the diminished fluorescence signal of NeuN indicated neuronal loss or injury. Analysis of DLAT expression showed that its fluorescence intensity in the HIBD group was generally lower than that in the Sham group. Importantly, the downregulation of DLAT expression correlated with the decreasing trend of the NeuN signal. In regions with increased IBA-1 or GFAP signals, the co-localized DLAT signal also demonstrated a declining trend, suggesting that DLAT downregulation may occur across multiple cell populations.

**Figure 4:**
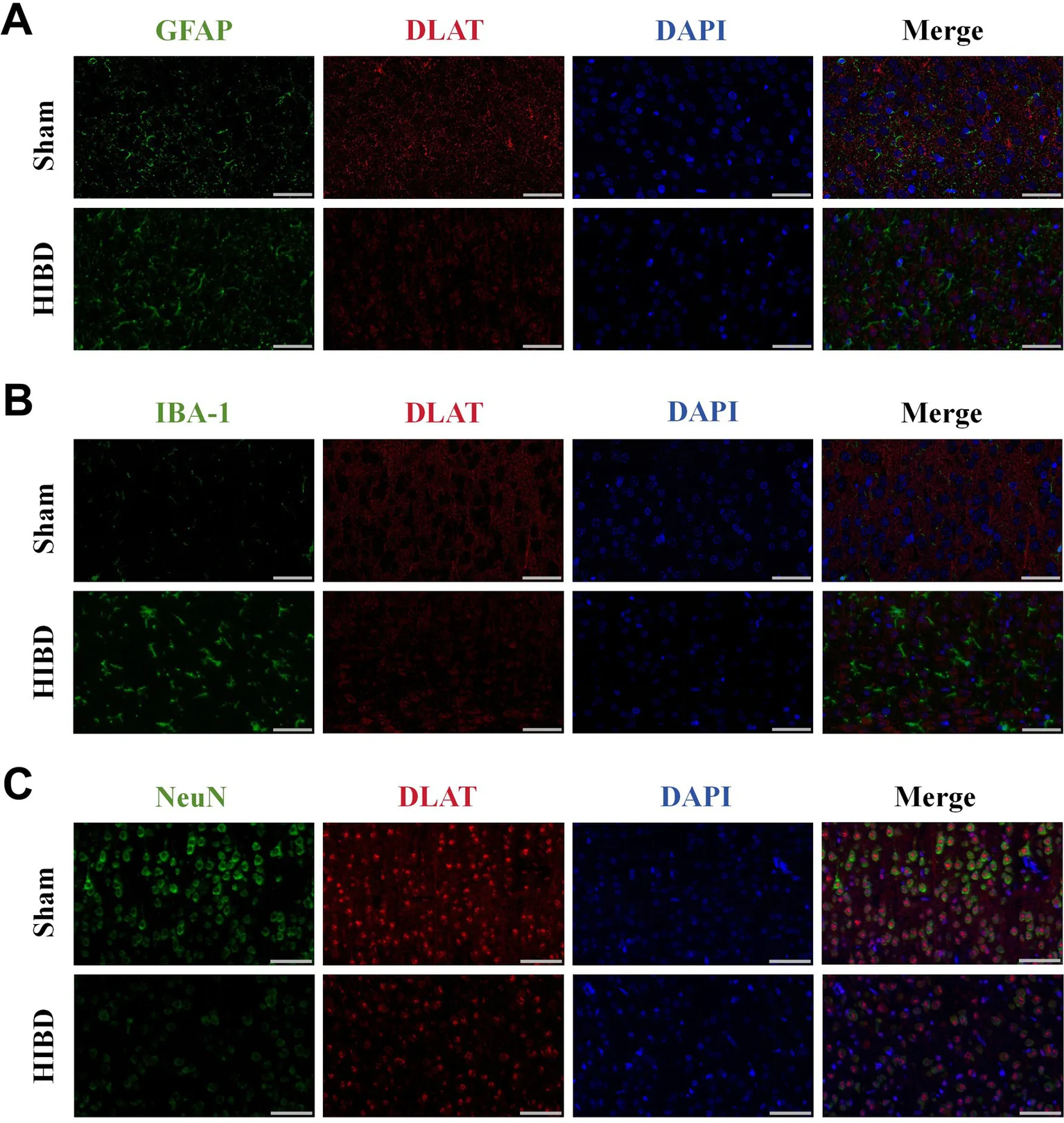
Changes of immunofluorescence expression in nerve cells and DLAT after HIBD. **(A–C)** Representative IF staining images of IBA-1/DLAT, GFAP/DLAT, and NeuN/DLAT in the cerebral cortex of P14 after HIBD modeling. Scale bar = 50 μm.

### 3.5 Alterations in the expression levels of cuproptosis-related markers after HIBD

Abnormal expression of cuproptosis-related proteins is a key molecular feature of cuproptosis. WB results indicated that, compared to the Sham group, the protein expression level of FDX1—an upstream regulator of cuproptosis—was significantly upregulated in the ischemic brain tissues of rats in the HIBD group (Figure 5). At the same time, the expression of HSP70, a stress-protective molecule, also increased, potentially reflecting a compensatory response of cells to injury caused by copper overload. Conversely, proteins involved in mitochondrial metabolism and lipoylation modification displayed a downward trend. The decreased expression of LIAS may indicate impaired lipoylation modification and mitochondrial respiratory chain function, while reduced expression of lipoylated dihydrolipoamide transacetylase (Lip-DLAT) suggests that inhibition of mitochondrial metabolic pathways could exacerbate the cuproptosis process (Figure 5). Furthermore, the significant downregulation of ACO2 may be linked to mitochondrial disruption resulting from an imbalance in copper metabolism (Figure 5). Collectively, these findings demonstrate a significant remodeling of the expression profile of cuproptosis-related proteins in the brain tissues of rats following HIBD. This abnormal expression pattern may represent an important molecular mechanism underlying neuronal death induced by copper ion accumulation, further suggesting that cuproptosis is activated during HIBD.

**Figure 5:**
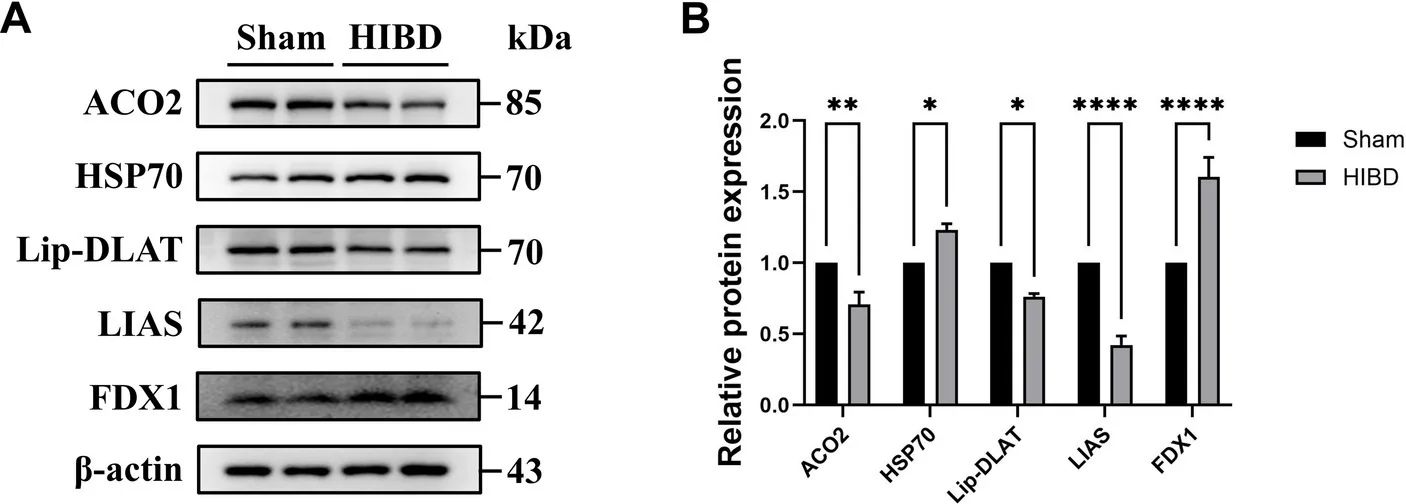
Relative expression of cuproptosis-related proteins in brain tissue after HIBD. **(A,B)** Representative western blot images and quantitation of ACO2, HSP70, Lip-DLAT, LIAS, and FDX1 at 24 h after HIBD modeling (normalized to β-actin). The values represent the mean ± SEM, *n* = 3. ****p < 0.0001, **p < 0.01, *p < 0.05 designate significant differences.

## 4 Discussion

This study established a neonatal rat HIBD model to characterize cuproptosis-associated molecular changes following HIBD. First, we confirmed the successful establishment of the model using methods such as LSCI, TTC staining, and brain water content measurement. Following this, HE staining and Nissl staining revealed a loose tissue structure, disordered cell arrangement, and a significant reduction in Nissl bodies in the cerebral cortex and hippocampal subregions of the HIBD group. These findings indicate that HIBD induces structural damage to brain tissue and impairs neuronal function. More importantly, TEM observations showed mitochondrial swelling, atrophy, and a reduction or complete disappearance of cristae structures in some cells of the HIBD group, along with dilation of the endoplasmic reticulum; however, no nuclear changes or chromatin condensation were observed.

These morphological changes appear distinct from the typical characteristics of apoptosis (cell shrinkage, plasma membrane blebbing, nuclear envelope rupture), necrosis (cell swelling, plasma membrane rupture, chromatin condensation), and autophagy (plasma membrane blebbing, autophagic vesicles, cytoplasmic exhaustion) (16, 17), and are suggestive of cuproptotic-like alterations (18, 19). Previous studies have shown that excessive copper inhibits the activity of mitochondrial respiratory chain complexes, disrupts the site-specificity of the mitochondrial electron transport chain, and results in mitochondrial dysfunction (20). Mitochondrial dysfunction represents a well-recognized feature across multiple forms of brain injury and is also a core hallmark of cuproptosis, raising the possibility of shared mitochondrial-centered pathological mechanisms in neonatal HIBD. This reasoning receives indirect support from studies in other cerebral-injury models and mechanistic reviews (21, 22).

Mitochondria are the main organelles for oxidative phosphorylation, regulating cellular redox homeostasis and metabolic status (23). The production of free radicals by the respiratory chain is regulated by various factors. This can impair mitochondrial bioenergetic synthesis by decreasing mitochondrial membrane fluidity and altering the activity of membrane-bound proteins (24, 25). *In vitro* experiments have confirmed that copper exposure influences cell viability and morphology in a dose-dependent manner. This exposure results in elevated free radical levels and reduced antioxidant enzyme activity, ultimately leading to mitochondrial oxidative stress and cell death (26, 27). The results of this study indicated that the copper ion content and ROS levels in the brain tissues of rats in the HIBD group were significantly elevated, while the levels of GSH and GPX4 were significantly reduced. Mechanistically, GSH serves a dual role in regulating copper-related oxidative stress. First, as a copper chaperone, GSH can directly bind excess cuprous ions to form stable complexes that are subsequently excreted from cells. Second, as a vital coenzyme for GPX4, GSH is involved in the clearance of intracellular ROS and lipid peroxides, thereby helping to maintain redox balance (28). During cuproptosis, oxidative stress is linked to an imbalance in copper ion homeostasis and dysfunction of the antioxidant system. Multiple studies have shown that copper chelator treatment can increase GSH levels, enhance antioxidant enzyme activity, and significantly reduce oxidative damage caused by excess copper (14, 29). In addition, inhibiting CTR1 expression can also significantly reduce copper-mediated oxidative stress responses (30, 31). Under HIBD conditions, the depletion of GSH and the reduction of GPX4 impair the cellular antioxidant capacity. This leads to an abnormal accumulation of ROS and exacerbates cuproptosis, suggesting that oxidative stress imbalance may occur alongside copper-related molecular perturbations after HIBD.

Protein lipoylation is a highly conserved post-translational modification, primarily functioning to regulate the activity of mitochondrial metabolic enzymes. This regulation is crucial for maintaining TCA cycle homeostasis and cellular energy metabolism (32). Only five mitochondrial proteins in human cells have been clearly identified as lipoylation targets, among which DLAT has been confirmed to be a key target of the cuproptosis pathway (21). To investigate the potential connection between neural cells and cuproptosis during HIBD, we analyzed the correlation between DLAT expression distribution and different cell types. The results indicated that neuroglial activation, neuronal loss, and a universal downregulation of DLAT expression occurred simultaneously in brain tissues following HIBD. Notably, DLAT reduction appeared to parallel the declining trend of neuronal markers, which may point toward a potential contribution of DLAT dysfunction to neuronal injury post-HIBD. This study proposes that HIBD may induce mitochondrial metabolic stress, leading to decreased expression or dysfunction of DLAT in neurons, thereby disrupting lipoylation homeostasis. Given the critical role of DLAT in the cuproptosis pathway, we speculate that this dysfunction could impair neurons’ ability to regulate copper ion metabolism, and may confer susceptibility to DLAT-associated cuproptosis-like molecular events, potentially aggravating neuronal injury. Importantly, robust glial activation was observed in the HIBD group, while obvious DLAT signal alterations were less prominent in activated glia. These observations hint that glial cells may predominantly contribute to neuroinflammatory responses following HIBD.

Several cerebral ischemia-related cuproptosis genes, including FDX1, LIAS, and DLAT, have been identified through bioinformatics analysis (33). Among them, FDX1 serves as both an upstream regulator of protein lipoylation and a key modulator of copper-induced cell death. On the one hand, FDX1 initiates free radical chain reactions catalyzed by LIAS by providing electrons to specifically biosynthesize lipoic acid cofactors (34), and can directly bind LIAS to regulate the lipoylation of downstream proteins (35). Conversely, copper ions that enter the mitochondria are reduced by FDX1 to more reactive cuprous ions. These cuprous ions then bind to Lip-DLAT, inducing its oligomerization and impacting the metabolic balance of the TCA cycle (36). In addition, excessive copper leads to massive loss of ISC proteins and increased expression of heat shock proteins, triggering cellular proteotoxic stress (37, 38). In this study, we observed that the expression of FDX1 and HSP70 was upregulated, while the expression of LIAS, ACO2, and Lip-DLAT was downregulated in the injured brain tissues of the HIBD group. The increase in FDX1 may facilitate the redistribution and activation of copper in mitochondria, thereby promoting the cuproptosis process (10). Elevated HSP70 may be a compensatory stress response to alleviate protein misfolding under copper reduction stress (39). Both LIAS and ACO2 are ISC proteins, suggesting impaired ISC biosynthesis, which may exacerbate mitochondrial dysfunction (40). More importantly, the decreased level of Lip-DLAT (the lipoylated form of DLAT) may change it from a soluble, functional form into insoluble, toxic aggregates. This transformation could further exacerbate cuproptosis by inhibiting mitochondrial metabolic pathways (41).

We note that measurements were performed at two distinct post-insult time-points in this study. Assays for copper level, oxidative stress, western blot and TEM ultrastructural evaluation were conducted at 24 h, capturing acute molecular and mitochondrial alterations shortly after HIBD. Histological injury and DLAT immunofluorescence were examined at 7 days, reflecting sub-acute tissue damage and cellular protein distribution after sustained injury progression. These two sets of observations reflect different phases of HIBD pathology. Comprehensive temporal profiling of cuproptosis-related molecules across multiple time-points would further clarify the dynamic mechanistic cascade.

In summary, this study identifies multiple molecular and ultrastructural features reminiscent of cuproptosis that coincide with neuronal injury in the neonatal rat HIBD model. The proposed mechanism is as follows: HIBD disrupts cerebral copper ion homeostasis and mitochondrial function, leading to oxidative stress imbalance and abnormal lipoylation, which may generate cellular conditions permissive for neuron-specific cuproptosis-like molecular events and contribute to neural injury. Specifically, HIBD causes a significant increase in cerebral copper ion levels, along with mitochondrial ultrastructural damage and heightened oxidative stress, characterized by increased ROS levels and decreased GSH and GPX4 expression. At the molecular level, the upregulated expression of FDX1, a key regulator of cuproptosis, may facilitate copper ion reduction/activation and protein lipoylation. Conversely, the downregulated expression of ISC proteins such as LIAS and ACO2 indicates impaired mitochondrial metabolic function. Further studies indicated decreased Lip-DLAT levels, which is one of the reported molecular signatures associated with cuproptosis and may interfere with TCA-cycle function. Additionally, DLAT signals were readily detected in neuronal populations, implying neurons could be an important cellular context for cuproptosis-related molecular alterations after HIBD. These findings provide new observational insights into HIBD-associated molecular changes and raise the possibility that targeting cuproptosis-related pathways might represent a promising therapeutic direction for HIBD, which requires further functional validation.

It should be noted that alterations in ROS, GSH and GPX4 are not specific markers for cuproptosis. These changes reflect generalized oxidative stress and impaired antioxidant capacity and also represent key features of ferroptosis, which has been previously demonstrated in neonatal HIBD by our group (42, 43). Accordingly, concurrent ferroptosis cannot be excluded in the present model. Distinguishing cuproptosis from other regulated cell-death pathways requires further functional experimental validation.

This study describes a set of molecular and ultrastructural observations consistent with cuproptosis-like changes in a neonatal rat HIBD model using various methods. However, several limitations warrant consideration. First, while the age selection of the animal model aligns with the high incidence of clinical neonatal hypoxic–ischemic encephalopathy, species differences in brain development timing and metabolic characteristics between rodents and humans may limit the direct applicability of the research findings to clinical settings. Second, the study primarily focused on the DLAT-mediated cuproptosis pathway and did not systematically analyze other key targets within the same pathway such as (dihydrolipoamide succinyltransferase, DLST) and their specificity across different neural cell types. As a result, our understanding of the complete molecular landscape of cuproptosis in HIBD remains incomplete. Third, only total brain copper concentration was quantified in the present study, while mitochondrial or labile cytosolic copper was not assessed, which limits the ability to link elevated bulk tissue copper levels to the disruption of functionally lipoylated proteins within mitochondria. Finally, only compared Sham and HIBD groups were included in the present work, without functional interventions such as copper chelation or genetic manipulation of cuproptosis-regulating genes. Therefore, we cannot confirm a causal role of cuproptosis in HIBD. The observed molecular alterations may represent secondary nonspecific changes following ischemic brain injury. Furthermore, the interaction between cuproptosis and other cell death modalities, such as apoptosis and ferroptosis in HIBD, requires additional investigation. These limitations highlight clear directions for future research.

## Data Availability

The original contributions presented in the study are included in the article/supplementary material, further inquiries can be directed to the corresponding authors.
